# miR‐148b inhibits glycolysis in gastric cancer through targeting SLC2A1

**DOI:** 10.1002/cam4.1008

**Published:** 2017-04-24

**Authors:** Xiangfu Ding, Jingjing Liu, Tianzhou Liu, Zhiming Ma, Dacheng Wen, Jiaming Zhu

**Affiliations:** ^1^ Department of Thyroid SurgeryThe Second Hospital of Jilin UniversityChangchun130041China; ^2^ Department of Gastrointestinal SurgeryThe Second Hospital of Jilin UniversityChangchun130041China

**Keywords:** Gastric cancer, glycolysis, microRNA‐148b, SLC2A1

## Abstract

Although the molecular biology of GC has been well characterized, early diagnostic biomarkers and effective therapeutic options in gastric cancer are still under investigation. Here, we found that miR‐148b expression decreased in human gastric cancer tissues compared with matched adjacent nontumor tissues by q‐PCR analysis and in situ hybridization. Further investigation revealed that overexpression of miR‐148b limited glycolysis including glucose consumption, lactate production in gastric cancer cell lines BGC‐823 and MKN45. Bioinformatics prediction uncovered that a dedicated transporters solute carrier family 2 member 1 (SLC2A1), also called GLUT1, was the direct target of miR‐148b. The target effects were further confirmed by luciferase assay and western blot analysis. Besides, a reverse correlation was observed between relative SLC2A1 and miR‐148b expression in human GC tissues compared with matched adjacent nontumor tissues. Subsequently, SLC2A1 suppression by SLC2A1 siRNA or specific inhibitor restricted the reduced effects of glycolysis mediated by miR‐148b while SLC2A1 overexpression abrogated the effect of miR‐148b on glycolysis. Our findings provided new evidence of miR‐148b in GC development through restraining glycolysis, highlighting the role of miR‐148b as a new target for GC treatment.

## Introduction

Although the rate of gastric cancer (GC) has declined over the last 50 years, GC remains high morbidity and mortality among all malignant tumors. Reports showed that a total of 989,600 new cases and 738,000 deaths were estimated in 2008 1. Due to the absence of specific symptoms and early diagnostic biomarkers, gastric cancer is often diagnosed at an advanced stage, when the prognosis remains unfavorable.

A class of small (about 18–22 nucleotides) noncoding RNA species, known as microRNAs (miRNAs), act as oncogenes or tumor suppressor genes playing important roles in a wide range of biological process including tumor 2, 3, 4. Gastric cancer is a complex chronic disease characterized by uncontrolled proliferation, migration, invasion, and failure of apoptotic cell death. An increasing number of studies have detected abnormal expression of miRNAs and confirmed their role in its tumor development. For example, miR‐204, miR‐37, and 146a are downregulated in GC. Ectopic expression of miR‐204 inhibits GC cell viability while ectopic expression of miR‐37 and 146a suppressed migration and invasion of GC cells 5, 6, 7. Overexpression of miR‐17‐5p/20a inhibits cell apoptosis and accelerates GC cell cycle progression by targeting tumor suppressor gene TP53INP1 and the cyclin‐dependent kinase inhibitor p21 8. miR‐150 targets tumor suppressive gene EGR2, facilitating GC cell growth 9, 10. miR‐26a and miR‐212 inhibit proliferation of GC cells by directly repressing the expression of their targets FGF9 and RBP2 11. However, the complete patterns of miRNAs regulated and involved in GC remain to be fully elucidated.

miR‐148b is a member of miR‐148/152 family, which includes miR‐148a, miR‐148b, and miR‐152. It is reported that miR‐148a and miR‐152 were lowly expressed in gastric cancer 12. Interestingly, miR‐148b has the same “seed sequences” as miR‐148a and miR‐152, indicating miR‐148b may also function in GC. Indeed, miR‐148b is showed to be significantly downregulated in GC tissues and cell lines. miR‐148b can inhibit cell proliferation in vitro and in vivo and suppresses tumorigenicity in vivo by targeting the CCKBR 13. However, the other role of miR‐148b in GC remained unknown and whether miR‐148 had other targets still need investigation.

Glucose is an essential source of energy for mammalian cells, and also used as a substrate in protein and lipid synthesis. Given its hydrophilic nature, glucose must be transported into the cell by dedicated transporters; these are encoded by genes known collectively as the facilitative glucose transporter gene family (GLUT). There are 13 known members of the GLUT family. SLC2A1 is also called GLUT1. Cancer cells, which proliferate at a greater rate, thus require more energy than normal cells.

In this study, we found significant downregulation of miR‐148b in 48 cases of gastric cancer tissues compared with matched nontumor counterparts by real‐time RT‐PCR analysis. We further confirmed an obvious expression decrease of miR‐148b in gastric cancer tissues by in situ hybridization. Moreover, the expression of miR‐148b was demonstrated to be associated with tumor grade, with even lower expression in high‐grade tumor. Glycolysis including glucose consumption, lactate production were both inhibited by miR‐148b overexpression in gastric cancer cell line BGC‐823 and MKN45, which were further strengthened under hypoxia condition. miR‐148b also inhibit cell growth and cell invasion. SLC2A1 was predicted to be the target of miR‐148b. The expression of SLC2A1 and miR‐148b in vivo showed a negative correlation. miR‐148b cannot further reduce the lactate production and glucose consumption when SLC2A1 was knocked down. SLC2A1 overexpression could abrogate the effect of miR‐148b on glycolysis. These findings provided important evidence that miR‐148b could suppress glycolysis in gastric cancer through directly targeting SLC2A1, further suggesting miR‐148b may be a potential biomarker and therapeutic target in gastric cancer treatment.

## Material and Methods

### Clinical specimens

Gastric cancer specimens, including adjacent nontumor tissues, were obtained from the Second Hospital of Jilin University. The Institutional Ethics Committee approved the study protocol and use of clinical specimen. Written, voluntary, informed consent was taken from all the patients. The specimens were obtained after surgical resection, immediately frozen, and stored in liquid nitrogen. The tumor characteristics information are summarized in Table S1 and ethical proof is provided in Table S2. Histological grade was determined blindly by two pathologists. The samples were classified into three histological grades including well‐differentiated grade I, moderately differentiated grade II, and poorly differentiated grade III according to Edmondson grading methods. Low grade was well‐to‐moderately differentiated (grade I, I‐II, and II). High grade was poorly differentiated (grade II‐III and III).

### Cell lines

All cell lines were obtained from the American Type Culture Collection and cultured according to standard mammalian tissue culture protocols and sterile technique. Human gastric cell lines BGC‐823 and MKN45 were maintained in DMEM media (Invitrogen, Carlsbad, CA), and supplemented with 10% (*v*/*v*) fetal bovine serum, 100 U/mL penicillin, and 100 mg/mL streptomycin. Cell culture was conducted at 37°C in a humidified 5% CO2 incubator. Cell transfection was performed with a lipofectamine 2000 reagent following the manufacturer's protocol. miR‐148b mimic, inhibitor, and siRNA targeting SLC2A1 (ON‐TARGETplu) were purchased from ThermoFisher and GE Dharmacon, respectively.

### Quantitative RT‐PCR

Total RNA was isolated from tissues and cell lines using the TRizol regent (Invitrogen), and reverse‐transcribed into cDNA. Expression of miR‐148b was detected by quantitative RT‐PCR‐based TaqMan^®^ MicroRNA assay (Applied Biosystems, Foster City, CA) followed by manufacturer's instructions. U6 snRNA was used as an endogenous control. The miRNA levels were determined using the 7500 Fast System SDS software (Applied Biosystems). The ddCt algorithm was used to calculate the relative quantification.

### In situ hybridization

In situ hybridization was performed using the locked nucleic acid (LNA). The digoxigenin‐labeled LNA probes (Exiqon, Vedbaek, Denmark) were used to detect miR‐148b. The probes were visualized using a horseradish peroxidase (HRP)‐conjugated anti‐digoxigenin antibody (Abcam, Cambridge, USA) and enzymatically reacted with 3,3′‐diaminobenzidine (DAB) substrate. The nuclei were stained using hematoxylin. The signal was evaluated by assessing staining intensity using a BX51 microscope (Olympus, Tokyo, Japan).

### Immunohistochemistry

Immunohistochemistry was performed according to standard protocols. The tissue sections were incubated with anti‐SLC2A1 antibody (Sigma‐Aldrich, 1:100 dilution) for 3 h. After incubation with the horseradish peroxidase‐conjugated secondary antibody (1:100 dilution), the signal was visualized using 3,3′‐diaminobenzidine (DAB) substrate. The sections were counterstained with hematoxylin and the staining was observed by BX51 microscope (Olympus, Tokyo, Japan).

### Lactate production, glucose consumption, and ATP assay

Cells were changed with fresh medium at 24 h after microRNA transfection. Cells were further cultured for 24 h, then medium were collected for measurement of lactate and glucose using lactate assay kit (Sigma‐Aldrich) and glucose assay kit (Sigma‐Aldrich). ATP levels were measured using ATP kit (PerkinElmer).

### Cell growth assay

Cell growth assay was carried out using BrdU Cell Proliferation Chemiluminescent Assay Kit (Cell Signaling) in 96‐well plate. Cells were incubated with BrdU for 4 h. A wavelength of 430 nm was measured using a Multiskan^™^ FC Microplate Photometer (Thermo Scientific).

### Cell invasion assay

Cells were plated in a serum‐free medium after transfection to the top of 8‐μm transwell inserts (BD Biosciences) in 24‐well companion plates (BD Biosciences) containing 10% FBS. After 24 h, cells were fixed with methanol and stained with crystal violet (Invitrogen). Cells on the top of inserts were removed using cotton swabs and migrated cells were dissociated in a Triton‐X100 buffer from the bottom side of inserts and read at 595 nm wavelength.

### Luciferase assay

Forward primer 5′‐CCCTCGAGGTCGCCCCAGATCACCAGC‐3′ and reverse primer 5′‐ GCTCTAGAGACAAATATCTTTGGTGT‐3′ were used to clone the 3′UTR of SLC2A1 into pmirGLO vector to build the WT‐3′UTR reporter vector. 5′‐TCCTTCGAAATAGGGGCCACACTATTACCAT‐3′ and 5′‐CTATTTCGAAGG AGACTAGAACCCGG‐3′ were further used to clone mutant 3′UTR of SLC2A1. Dual‐luciferase reporter assay was performed in BGC‐823 cells following manufacturer's instructions (Promega, Madison, WI). Cells were cotransfected with reporter vector, along with miR‐148b mimics or control miRNA. Forty‐eight hours after transfection, cells were rinsed with PBS, and performed with dual‐luciferase assay. Luciferase activity was measured using a Victor Luminometer (Perkin Elmer, Waltham, MA). The firefly luciferase activity was normalized using cotransfected Renilla luciferase for transfection efficiency. All experiments were performed in triplicate.

### Immunoblotting

Cells were lysed in a RAPI lysis buffer and solubilized in SDS loading buffer. Equal amount of protein extracts were separated a polyacrylamide gel and transferred to a nitrocellulose membrane (Amersham Biosciences). Protein expression was analyzed using standard procedures for western blot analysis. The membranes were incubated with a primary antibody (anti‐SLC2A1, Santa Cruz Biotechnology; anti‐*β*‐actin, Santa Cruz Biotechnology). After incubation with appropriate horseradish peroxidase‐conjugated secondary antibody (Santa Cruz Biotechnology), the membranes were treated with an enhanced chemiluminescence reagent (Thermo scientific, Dreieich, Germany), and exposed to X‐ray film (Kodak, Rochester, USA).

### Statistical analysis

Data were analyzed using the SPSS software (SPSS Inc., Chicago, IL). Quantitative data were presented as the mean ± the standard deviation. Statistical differences between groups were determined by the Student's t‐test. Differences were considered significant when *P* < 0.05. Differences between miR‐148b expression in tumor tissues and adjacent nontumor tissues were analyzed by the Wilcoxon matched pairs test.

## Results

### miR‐148b is downregulated in gastric cancer

**Figure 1:**
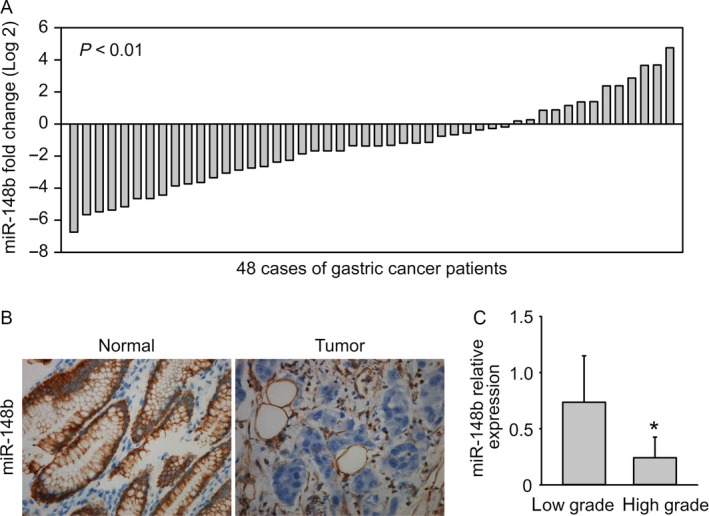
miR‐148b is downregulated in gastric cancer. (A) miR‐148b is downregulated in gastric cancer tissues compared to adjacent nontumor tissues, examined by q‐PCR. (B) miR‐148b is downregulated in gastric cancer examined by in situ hybridization. (C) miR‐148b is correlated with GC tumor grade.

A number of miRNAs are involved in the regulation of gastric cancer. Through analyzing the samples of 48 cases of gastric cancer tissues compared with adjacent nontumor tissues (Table S1), we found that the expression of miR‐148b was significantly downregulated in gastric cancer tissues (Fig. 1A). miR‐148b in the 48 paired tissues were also analyzed by in situ hybridization staining. The results are in line with q‐PCR analysis, with miR‐148b decreased markedly in tumor tissues (Fig. 1B). q‐PCR analysis was further carried out to test whether miR‐148b expression is associated with tumor grades. miR‐148b expression was even lower in high‐grade gastric cancer tumors than low‐grade gastric cancer tumors (Fig. 1C).

### miR‐148b inhibits glycolysis in gastric cancer

**Figure 2:**
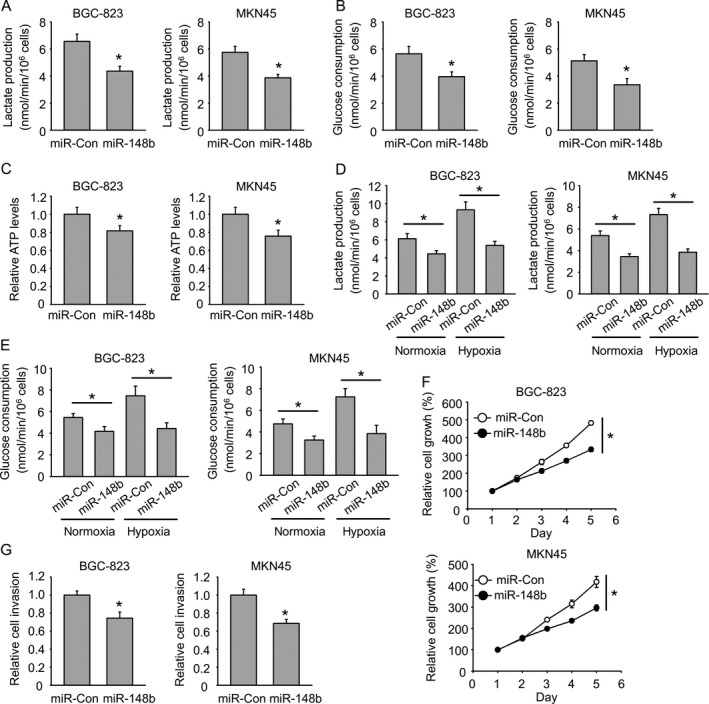
miR‐148b inhibits glycolysis in gastric cancer. (A) miR‐148b inhibits lactate production. (B) miR‐148b inhibits glucose consumption. (C) miR‐148 reduces ATP levels. (D) miR‐148b inhibits hypoxia‐induced increase of lactate production. (E) miR‐148b inhibits hypoxia‐induced increase of glucose consumption. (F) miR‐148b reduces cell growth. (G) miR‐148b reduces cell invasion.

**Figure 3:**
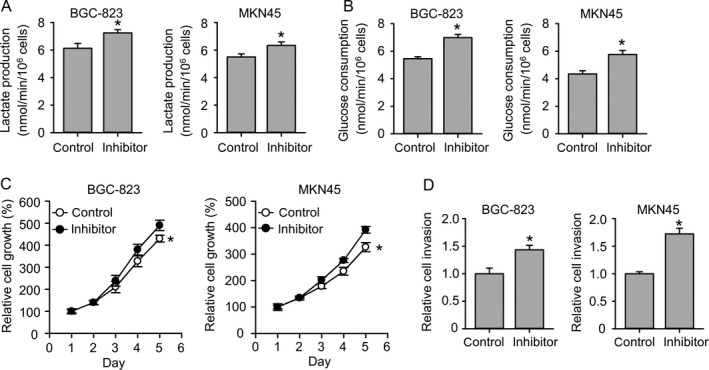
miR‐148b inhibitor promotes glycolysis in gastric cancer. (A) miR‐148b inhibitor promotes lactate production. (B) miR‐148b inhibitor promotes glucose consumption. (C) miR‐148b inhibitor promotes cell growth. (D) miR‐148b inhibitor promotes cell invasion.

To examine the potential role of miR‐148b in gastric cancer development, human gastric cancer cell line BGC‐823 and MKN45 were adopted. Cells were transfected with miR‐148b mimics and then cultured for 48 h. Then, concentration of glucose and lactic acid in the culture supernatant was measured using D‐Glucose kit or L‐Lactic acid kit. Both cells showed decreased lactate production and glucose consumption after miR‐148b transfection (Fig. 2A and B). Relative ATP levels in both cells were also shut down by miR‐148b (Fig. 2C). Next, lactate production and glucose consumption under normoxia and hypoxia were also measured, respectively. Hypoxia promoted higher lactate production and glucose consumption than normoxia, whereas miR‐148b totally abrogated those effects (Fig. 2D and E). The reduced glycolysis rate and ATP production made us further identify the effects of miR‐148b on cell growth. The cell growth was significantly suppressed by miR‐148b in both cell lines (Fig. 2F). The cell invasion was also restricted by miR‐148b (Fig. 2G). We further investigated whether miR‐148b inhibitor has the opposite role. miR‐148b inhibitor increased lactate production and glucose consumption in both BGC‐823 cells and MKN45 cells (Fig. 3A and B). Consistent with increased glycolysis, we also observed increased cell proliferation and invasion upon miR‐148b inhibitor transfection (Fig. 3C and D). These results further support the tumor suppressor role of miR‐148b in gastric cancer.

### miR‐148b target SLC2A1 in gastric cancer cells

**Figure 4:**
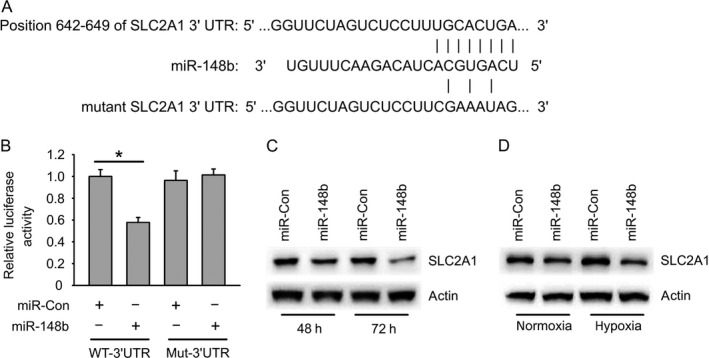
miR‐148b target SLC2A1 in gastric cancer cells. (A) Schema of binding site of miR‐148b in SLC2A1 3′UTR region. (B) SLC2A1 3′UTR luciferase activity regulated by miR‐148b in BGC‐823 cells. (C) miR‐148b reduces endogenous SLC2A1 protein expression in BGC‐823 cells. (D) miR‐148b reduces hypoxia‐induced increase of SLC2A1 protein levels in BGC‐823 cells.

To explore the role of miR‐148b in glycolysis, a computational analysis was performed in order to identify putative target genes for miR‐148b. Bioinformatics analysis revealed that miR‐148b target the sequences in position of 642‐649 in 3′‐UTR of SLC2A1 (Fig. 4A). SLC2A1 3′‐UTR sequence was cloned downstream of the luciferase reporter gene. We also cloned mutant SLC2A1‐3′UTR including several mutation in miR‐148b‐binding site which could not be targeted by miR‐148b (Fig. 4A). Luciferase assay showed that miR‐148b reduced the SLC2A1‐3′UTR but not SLC2A1‐3′UTR‐mut luciferase levels in BGC‐823 cells (Fig. 4B). miR‐148b also lowered the protein levels of SLC2A1 72 h after transfection but not 48 h (Fig. 4C). The suppression effect was further strengthened under hypoxia (Fig. 4D). These data indicate that miR‐148b modulated SLC2A1 expression by directly targeting the 3′‐UTR of SLC2A1 mRNA.

### SLC2A1 is upregulated in gastric cancer cells and promote glycolysis

**Figure 5:**
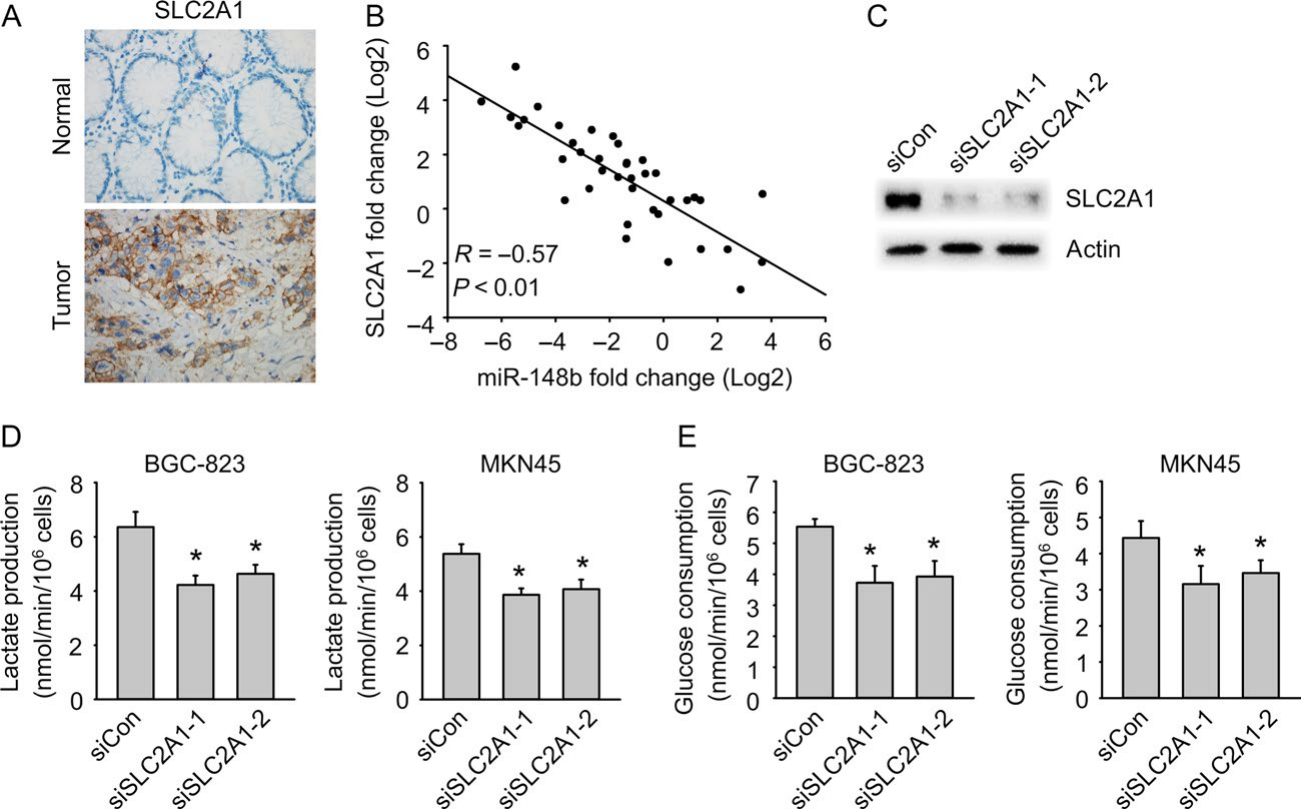
SLC2A1 is upregulated in gastric cancer cells and promote glycolysis. (A) SLC2A1 is increased in gastric cancer by immunohistochemistry. (B) SLC2A1 is negatively correlated with miR‐148b. (C) Immunoblot analysis of the knockdown efficiency of SLC2A1 in BGC‐823 cells treated with control siRNA (SiCon) or SLC2A1 siRNA (siSLC2A1‐1 or siSLC2A1‐2) for 24 h. (D) SLC2A1 knockdown reduced glycolysis (lactate production). (E) SLC2A1 knockdown reduced glycolysis (glucose consumption).

To examine the role of SLC2A1 in gastric cancer tumor tissues, the protein levels of SLC2A1 expression in tumor tissues and matched normal tissues were detected by immunohistochemistry staining. SLC2A1 was highly expressed in tumor tissues (Fig. 5A). Since SLC2A1 was shown to be targeted by miR‐148b, we then wondered whether the in vivo expression of SLC2A1 would be connected to miR‐148b. A reverse correlation was observed between relative SLC2A1 and miR‐148b expression (Fig. 5B). To further find out the in vivo role of SLC2A1 with glycolysis, we synthesized two siRNAs targeting SLC2A1, which both displayed obvious knockdown efficiency (Fig. 5C). Lactate production and glucose consumption were clearly reduced in BGC‐823 and MKN45 cells when SLC2A1 was knocked down (Fig. 5D and E).

### Overexpression of SLC2A1 abrogates the effect of miR‐148b on glycolysis

**Figure 6:**
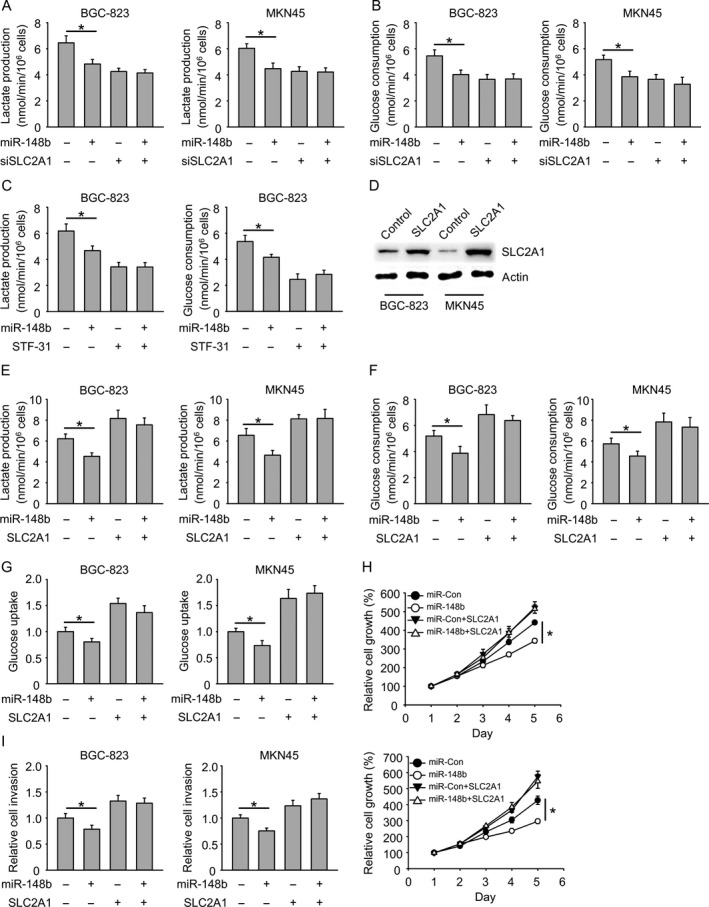
Overexpression of SLC2A1 abrogates the effect of miR‐148b on glycolysis. (A) miR‐148b cannot reduce glycolysis (lactate production) further when SLC2A1 is knocked down. (B) miR‐148b cannot reduce glycolysis (glucose consumption) further when SLC2A1 is knocked down. (C) miR‐148b cannot reduce glycolysis further when SLC2A1 inhibitor STF‐31 is added. (D) Immunoblot analysis of SLC2A1 overexpression by lentivirus‐mediated overexpression. (E–G) SLC2A1 rescues miR‐148b mediated reduction of glycolysis (lactate production, glucose consumption, glucose uptake). (H) SLC2A1 rescues miR‐148b mediated reduction of cell growth. (I) SLC2A1 rescues miR‐148b mediated reduction of cell invasion.

To further confirm if SLC2A1 was the target of miR‐148b, the effect of miR‐148b on glycolysis was examined in both SLC2A1‐knockdown BGC‐823 and MKN45 cells. miR‐148b cannot further reduce the lactate production and glucose consumption when SLC2A1 was knocked down (Fig. 6A and B). Consistent with that, miR‐148b cannot further reduce glycolysis in BGC‐823 cells when SLC2A1 inhibitor STF‐31 was added (Fig. 6C and Figure S1A). Furthermore, SLC2A1 was overexpressed in BGC‐823 and MKN45 cells mediated by lentivirus. Overexpression was confirmed by western blot analysis (Fig. 6D). SLC2A1 overexpression rescued miR‐148b mediated reduction of glycolysis including lactate production, glucose consumption, and glucose uptake (Fig. 6E–G and Figure S1B and C). The cell growth and invasion was also rescued by SLC2A1 overexpression in both cell lines (Fig. 6H and I). These results indicated that SLC2A1 overexpression could abrogate the effect of miR‐148b on glycolysis.

## Discussion

miR‐148b was previously reported to be associated with tumorigenicity. The tumor volume at the time of death in nude mice injected with miR‐148b mimics‐transfected cells was lower than that of mice injected with NC or MGC‐803 cells 13. Moreover, the mean tumor weight was also markedly lower in the group with miR‐148b mimics compared to the NC and MGC‐803 groups 13. Our study found that miR‐148b was associated with tumor grade in human. Given the expression of miR‐148b was commonly downregulated in GC tissues, the expression of miR‐148b was even lower in high‐grade GC than low‐grade GC. These results were not only consistent with the above data but also highlighted the biomarker role of miR‐148b in tumor progression and prognosis.

It was reported that rapidly proliferating ascites tumor cells consume glucose at a surprisingly high rate compared to normal cells and secrete most of the glucose‐derived carbon as lactate rather than oxidizing it completely, a phenomenon known as the “Warburg effect”^14^, ^15^. Glucose transporters, in particular SLC2A1, is a rate‐limiting transporter for glucose uptake, and its expression correlates with anaerobic glycolysis 16. Thus, SLC2A1 have become a target of interest in cancer research 17. Here, we identified miR‐148b regulated GC through directly inhibiting glycolysis. Ectopic expression of miR‐148b in GC cell line BGC‐823 and MKN45 inhibited the ability of glucose uptake and lactate production as well as ATP production, further impairing cell growth and invasion. Moreover, knockdown of SLC2A1 by siRNA or specific inhibitors, which blocked glucose uptake by tumor cells, cannot further reduce the ability of glycolysis. In contrast, ectopic expression of SLC2A1 abolished the effects mediated by miR‐148b.These results suggested that miR‐148b at least function through modulating glycolysis in GC. miR‐148b is in the same family with miR‐148b and miR‐152. We also compared their roles in gastric cancer glycolysis. miR‐148a inhibits gastric cancer glycolysis as miR‐148b, while miR‐152 has little effect on glycolysis (Figure S2A and B), suggesting that miR‐152 may have different target genes. Indeed, we found that miR‐152 only leads to slight reduction of SLC2A1 protein levels (Figure S2 C and D). In addition, miR‐148a is also reduced in gastric cancer, but with less extent compared to miR‐148b (Figure S2E).

Defining the role of miRNAs in gastric cancer and their functional mechanism is important for improving the sensitivity of diagnosis and developing efficient gastric cancer treatments. A large number of miRNAs have been shown to be associated with tumor type, tumor stage, and patient survival and therefore may be developed as novel diagnostic or prognostic markers 18, 19, 20, 21, 22. Additionally, circulating miRNAs found in the blood of patients constitute the most promising type of miRNAs for clinical use for their convenience and fast in tracking pathological progression, predicting prognosis 23, 24, 25, 26. However, the precise mechanism of each miRNA is not well known. Each miRNA modulates the expression of hundreds of genes, and it is indicated that miRNAs may act in a network‐type fashion to mediate the expression of genes. Although increasing numbers of miRNAs appear, it is necessary to take into consideration of utilization of miRNAs combined.

Taken together, our study provides a new perspective of miR‐148b in GC development through inhibiting glycolysis in GC cells. miR‐148b function directly targeting glucose transporter SLC2A1, may strengthen the role of miR‐148b as a target for early clinical diagnosis and treatment for gastric cancer.

## Conflict of Interest

None declared.

## Supporting information


**Figure S1.** (A) SLC2A1 protein levels corresponding to Figure 6C. (B) SLC2A1 protein levels corresponding to Figure 6E–G. (C) miR‐148b levels corresponding to Figure 6E–G.

**Figure S2.** (A) Lactate production in BGC‐823 cells after transfection of miR‐148b, miR‐148a, or miR‐152. (B) Glucose consumption in BGC‐823 cells after transfection of miR‐148b, miR‐148a, or miR‐152. (C) SLC2A1 3′UTR luciferase activity regulated by miR‐148b, miR‐148a, or miR‐152 in BGC‐823 cells. (D) SLC2A1 protein levels in BGC‐823 cells after transfection of miR‐148b, miR‐148a, or miR‐152. (E) miR‐148a is downregulated in gastric cancer tissues compared to adjacent nontumor tissues, examined by q‐PCR.

**Table S1.** Clinicopathologic characteristics of gastric cancer patients.
