# Supplementary figures and images for: miR‐148b inhibits glycolysis in gastric cancer through targeting SLC2A1
Source: Cancer Med. 2017 Apr 24;6(6):1301–10. doi: 10.1002/cam4.1008 (PMC5463086; doi:10.1002/cam4.1008)

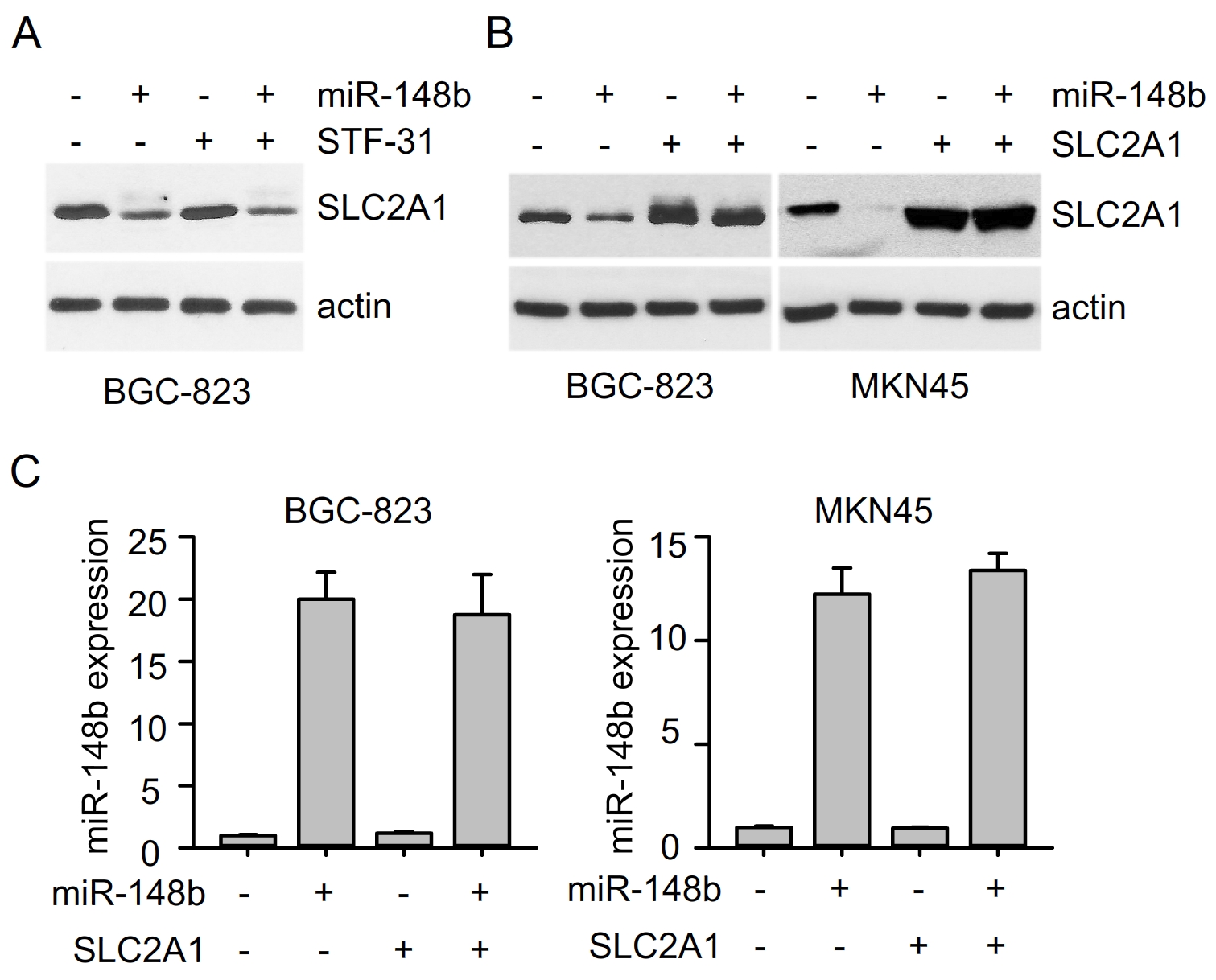

Supplement: Supplementary file 1 — Figure S1. (A) SLC2A1 protein levels corresponding to Figure 6C. (B) SLC2A1 protein levels corresponding to Figure 6E–G. (C) miR‐148b levels corresponding to Figure 6E–G. [file CAM4-6-1301-s001.jpg]

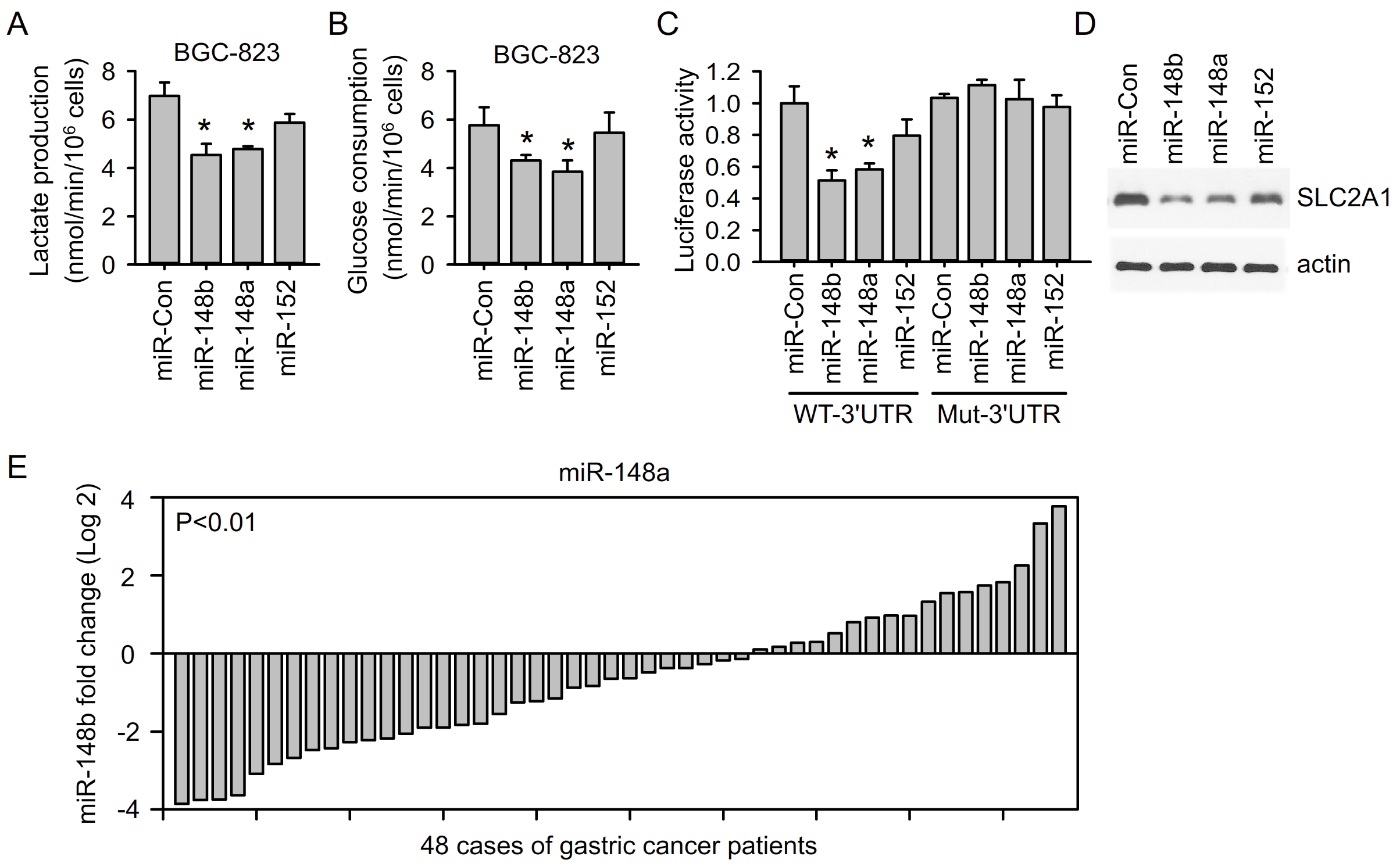

Supplement: Supplementary file 2 — Figure S2. (A) Lactate production in BGC‐823 cells after transfection of miR‐148b, miR‐148a, or miR‐152. (B) Glucose consumption in BGC‐823 cells after transfection of miR‐148b, miR‐148a, or miR‐152. (C) SLC2A1 3′UTR luciferase activity regulated by miR‐148b, miR‐148a, or miR‐152 in BGC‐823 cells. (D) SLC2A1 protein levels in BGC‐823 cells after transfection of miR‐148b, miR‐148a, or miR‐152. (E) miR‐148a is downregulated in gastric cancer tissues compared to adjacent nontumor tissues, examined by q‐PCR. [file CAM4-6-1301-s002.jpg]

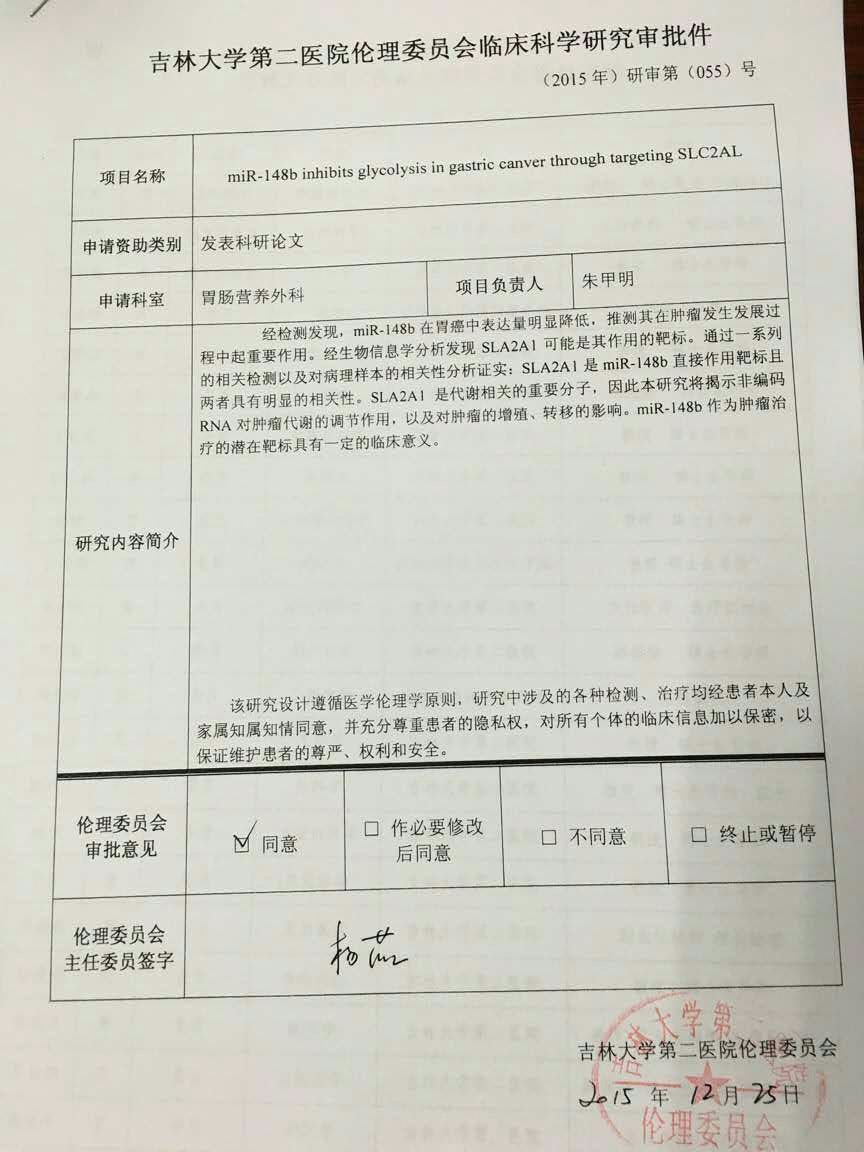

Supplement: Supplementary file 3 — Table S1. Clinicopathologic characteristics of gastric cancer patients. [file CAM4-6-1301-s003.jpg]
