# Supplementary material for: miR‐148b inhibits glycolysis in gastric cancer through targeting SLC2A1
Source: Cancer Med. 2017 Apr 24;6(6):1301–10. doi: 10.1002/cam4.1008 (PMC5463086; doi:10.1002/cam4.1008)
Supplement: Supplementary file 4 [file CAM4-6-1301-s004.docx]

Supplemental figure 1.

A. SLC2A1 protein levels corresponding to Figure 6C.

B. SLC2A1 protein levels corresponding to Figure 6E-G.

C. miR-148b levels corresponding to Figure 6E-G.

Supplemental figure 2.

A. lactate production in BGC-823 cells after transfection of miR-148b, miR-148a or miR-152.

B. glucose consumption in BGC-823 cells after transfection of miR-148b, miR-148a or miR-152.

C. SLC2A1 3’UTR luciferase activity regulated by miR-148b, miR-148a or miR-152 in BGC-823 cells.

D. SLC2A1 protein levels in BGC-823 cells after transfection of miR-148b, miR-148a or miR-152.

E. miR-148a is downregulated in gastric cancer tissues compared to adjancent non-tumor tissues, examined by q-PCR.
